# Syndrome d’antidiurèse inappropriée néphrogénique

**DOI:** 10.11604/pamj.2019.32.210.6006

**Published:** 2019-04-29

**Authors:** Benabdellah Nawal, Hassan Izzedine, Intissar Haddiya, Yassamine Bentata

**Affiliations:** 1 Service de Néphrologie, CHU Mohammed VI, Oujda, Maroc; 2 Service de Néphrologie, Hôpital Privé des Peupliers, 75013 Paris, Paris, France

**Keywords:** Arginine vasopressine, récepteurs G couplés aux protéines, syndrome d’antidiurèse inappropriée néphrogénique, syndrome de sécrétion inappropriée d’hormone antidiurétique, récepteur V2 de la vasopressine, Arginine vasopressin, G-protein coupled receptors, nephrogenic syndrome of inappropriate antidiuresis, syndrome of inappropriate antidiuretic hormone secretion, vasopressin receptor type II

## Abstract

Les troubles de l'équilibre de l'eau représentent une pathologie fréquente dans notre pratique clinique. L'étude du récepteur V2R est importante pour la compréhension de la physiologie de l'équilibre hydrique et a servi de prototype pour la biologie des récepteurs G couplés aux protéines. Le syndrome d'antidiurèse inappropriée néphrogénique (NSIAD) est un syndrome de sécrétion inappropriée d'hormone antidiurétique (SIADH)-like avec un dosage plasmatique de vasopressine bas. La preuve du rôle du récepteur V2R et de l'aquaporine (AQP) dans le mode d'action de l'hormone antidiurétique (ADH) est venue de l'identification de mutations dans les gènes de ces protéines dans le diabète insipide néphrogénique et dans le syndrome NSIAD. Des mutations activatrices du V2R ont été retrouvées dans le NSIAD, à l'opposé des nombreuses mutations inactivatrices du V2R liées à l'X décrites dans le diabète insipide néphrogénique.

## Abstract (en)

Disorders of water balance are a disease commonly encountered in our clinical practice. Analysis of vasopressin receptor type II (V2R) is essential to understand the physiology of water balance and it is used as a biological prototype of G protein-coupled receptors (GPCRs). Nephrogenic syndrome of inappropriate antidiuresis (NSIAD) is a syndrome of inappropriate antidiuretic hormone secretion (SIADH) with low plasmatic vasopressin. The evidence on the role of V2 receptor and of aquaporin (AQP) in the mechanism of action for antidiuretic hormone (ADH) was based on the identification of protein gene mutations in patients with nephrogenic diabetes insipidus and NSIAD syndrome. V2R activating mutations were found in patients with NSIAD, contrasting with the numerous V2R inactivating mutations related to X-linked mutations described in patients with nephrogenic diabetes insipidus.

## Introduction

Le maintien d'un équilibre hydrique approprié est essentiel à la vie. L'homéostasie hydrique dépend de l'apport adéquat en eau, régi par un mécanisme de la soif intact, et par l'excrétion urinaire de l'eau libre, médiée par la sécrétion d'arginine vasopressine approprié (AVP), également connu sous le nom d'hormone antidiurétique (ADH) [1].

## Méthodes

Il s'agit d'une revue détaillée de la littérature sur les différentes bases physiopathologiques du syndrome d'antidiurèse inappropriée néphrogenique.

## Etat actuel des connaissances

**Physiologie:** l'AVP/ADH est sécrétée dans la circulation par la posthypophyse, en réponse à une augmentation de l'osmolalité ou à une baisse du volume circulant efficace. Au niveau rénal, l'AVP/ADH se lie à un récepteur couplé aux protéines G (RGCP), le récepteur V2 à la vasopressine (V2R) au niveau des tubes collecteurs, pour rendre la membrane cellulaire perméable à l'eau. Sous l'influence du gradient osmotique du sodium, l'eau est réabsorbée dans la cellule au pôle apical par l'aquaporine-2 (AQP2); et ressort de la cellule au pôle basolatéral par l'AQP-3 et l'AQP-4. La suppression de l'AVP/ADH restaure l'imperméabilité cellulaire. Ainsi, l'AVP/ADH exerce son action antidiurétique en se liant au récepteur V2R. La liaison du ligand active le V2R, en stimulant l'adénylate cyclase par l'intermédiaire de protéines Gs. L'augmentation de la concentration intracellulaire d'adénosine monophosphate (AMP) cyclique (AMPc) favorise le transfert de vésicules intracellulaires contenant le canal à eau AQP2 à la membrane apicale des cellules du canal collecteur, ce qui augmente la perméabilité à l'eau et induit une antidiurèse. L'étude du récepteur V2R a été importante pour la compréhension de la physiologie de l'équilibre hydrique et a servi de prototype pour la biologie des RGCPs. La preuve du rôle du récepteur V2R et de l'AQP est venue de l'identification de mutations dans les gènes de ces protéines dans le diabète insipide néphrogénique et dans le syndrome de sécrétion inappropriée d'hormone antidiurétique (SIADH).

**L'hyponatrémie:** le trouble électrolytique est le plus fréquent chez les personnes hospitalisées [2]. L'hyponatrémie légère (natrémie < 135 mmol par litre) survient chez 15 à 22% des patients et chez environ 7% des sujets ambulatoires [3]. L'hyponatrémie modérée (natrémie < 130 mmol par litre) survient chez 1 à 7% des patients hospitalisés [4, 5]. Il est important d'en faire le diagnostic à la fois à cause de sa potentielle morbidité et parce qu'elle peut être un marqueur d'une maladie sous-jacente. En effet, l'hyponatrémie sévère (natrémie < 125 mmol par litre), surtout lorsqu'elle est d'installation rapide (dans les 48 heures), entraine confusion, hallucinations, convulsions, coma, et un arrêt respiratoire conduisant à la mort. Des symptômes moins graves d'hyponatrémie comprennent la céphalée, une difficulté de concentration, des troubles de la mémoire, des crampes musculaires et une faiblesse; une dysgueusie a également été rapportée. Les patients présentant une hyponatrémie chronique peuvent être asymptomatiques, bien que certaines données suggèrent que les déficits neurologiques, responsable de chutes peuvent être plus fréquents chez les patients présentant une hyponatrémie chronique que chez les personnes à natrémie normale [6]. Le seuil de la natrémie à l'origine de complications neurologiques semble être plus élevé chez les femmes que chez les hommes [7]. Plus problématique sont les nausées et les vomissements qui sont à la fois un stimulus extrêmement puissant pour la libération d'AVP mais aussi qui peuvent résulter de l'hyponatrémie. Il est parfois difficile de déterminer si elles sont la cause ou la conséquence d'un cercle vicieux qui perpétue la sécrétion excessive d'AVP déclenché initialement par un autre stimulus. Dans ces cas, ces effets peuvent être évités ou corrigé complètement avec un traitement antiémétique [3].

**Les récepteurs G couplés aux protéines (RGCP):** les RGCP constituent la plus grande famille de récepteurs impliqués dans la transduction du signal et sont responsables de la régulation de plusieurs processus physiologiques [8]. De nombreuses maladies sont causées par des mutations des gènes de ces RGCP [9, 10]. Il s'agit de mutations « perte-de-fonction » inactivatrices (absence de liaison ligand-récepteur), ou inversement de mutations « gain-de-fonction » entrainant une activation constitutive du récepteur. Dans un certain nombre de RGCPs, des mutations activatrices ou inhibitrices ont été décrites sur différents sites d'un même récepteur traduisant le spectre clinique de la maladie, tel que le récepteur de la thyréostimuline (adénome de la thyroïde et l'hyperthyroïdie non-autoimmune familial versus l'hypothyroïdie congénitale), le récepteur de l'hormone lutéinisante (la testotoxicosis, les tumeurs testiculaires de Leydig versus les organes génitaux mâles ambigus) et le récepteur de l'hormone parathyroïdienne (chondrodysplasie métaphysaire de Jansen versus chondrodysplasie de Blomstrand) [9]. Il est très rare que des mutations portant sur un site unique d'acide aminé puissent transformer un récepteur sauvage soit en un récepteur actif ou inactif. Les seuls deux exemples cliniques portent sur les récepteurs du calcium (le récepteur calcium sensor) [11] et le récepteur V2R [12] (Tableau 1). Le motif DRY/H comprenant l'acide aspartique, l'arginine, la tyrosine ou l'histidine est un motif hautement conservé des GPCR [13]. L'arginine dans ce motif est invariante et des mutations faux-sens conduisent à des altérations de la fonction du récepteur comme des mutations dans la rhodopsine conduisant à une rétinite pigmentaire, et dans le récepteur de l'hormone de libération des gonadotrophines, conduisant à un hypogonadisme hypogonadotrophique. Le V2R, un RGCP de classe 1b, [10, 14] existe dans le plasma à l'état d'équilibre sous des conformations inactive et active [8]. La liaison du ligand déplace l'équilibre à l'état actif, ce qui permet un couplage avec les protéines G intracellulaires et l'activation d'effecteurs intracellulaires. Avec l'activation, le V2R est désensibilisé par phosphorylation par les kinases spécifiques des RGCPs. En conséquence, le recrutement de la bêta-arrestine par le récepteur phosphorylé termine le signal en bloquant l'interaction avec les protéines Gs et initie également l'internalisation du récepteur grâce à sa capacité à lier la clathrine et autres adaptateurs endocytiques [15]. En théorie, un tel gain-de-fonction pourrait également affecter de nombreux autres aspects de la biologie du V2R, y compris la phosphorylation, l'intériorisation, la régulation et le recyclage à la membrane cellulaire [12, 16].

**Tableau 1:** Maladies causées par des mutations des récepteurs G couplés aux protéines (RGCPs)

Récepteur	Mutation	Transmission	Maladie
**Opsines M**	Perte de fonction	Liée à l’X, TAR	Daltonisme
**Rhodopsine**	Perte de fonction	TAD, TAR	Rétinite pigmentaire
	Gain de fonction	TAD	Cécité nocturne congénitale
**V2R**	Perte de fonction	Liée à l’X	Diabète insipide néphrogénique
	Gain de fonction	Liée à l’X	Syndrome d’antidiurèse inappropriée néphrogénique
**ACTH**	Perte de fonction	TAR	Résistance à l’ACTH familiale
**LH**	Perte de fonction	TAR	Pseudohermaphrodisme male
	Gain de fonction	TAD	Puberté masculine précoce familiale
	Gain de fonction	Somatique	Tumeurs de Leydig sporadiques
**Ca2+ sensor**	Perte de fonction	TAD	Hypercalcémie hypocalciurique familiale
	Perte de fonction	TAR	Hyperparathyroïdie néonatale
	Gain de fonction	TAD	Hypocalcémie familiale
**Endotheline B**	Perte de fonction	Complexe	Maladie de Hirschsprung
**FSH**	Perte de fonction	TAR	Insuffisance ovarienne hypergonadotrope
**TSH**	Perte de fonction	TAR	Hypothyroïdie congénitale
	Gain de fonction	TAD	Hyperthyroïdie nonautoimmune familiale
	Gain de fonction	Somatique	Adénomes thyroïdiens hyperfonctionnels sporadiques
**TRH**	Perte de fonction	TAR	Hypothyroïdie centrale
**GHRH**	Perte de fonction	TAR	Déficience en hormone de croissance
**GnRH**	Perte de fonction	TAR	Hypogonadisme central
**Melanocortine 4**	Perte de fonction	Codominant	Obésité extrême
**PTH/PTHrP**	Perte de fonction	TAR	Chondrodysplasie de Blomstrand
	Gain de fonction	TAD	Chondrodysplasie métaphysaire de Jansen

RGCPs, récepteurs G couplés aux protéines; V2R, récepteur 2 de la vasopressine; ACTH, hormone corticotrope; LH, hormone lutéinisante; FSH, hormone folliculo-stimulante; TSH, thyréostimuline; TRH, hormone de libération de la thyrotropine; GHRH, hormone libérant l'hormone de croissance; GnRH, hormone de libération des gonadotrophines; PTH, parathormone; PTHrP, parathormone related peptide; TAR, transmission autosomique récessive; TAD, transmission autosomique dominante.

**Le syndrome d'antidiurèse inapproprié (SIAD):** le syndrome d'antidiurése inapproprié (SIAD) est un trouble hétérogène caractérisé par une hyponatrémie et un défaut de dilution des urines en l'absence d'insuffisance rénale ou de tout stimulus non osmotique identifiable connu pour entrainer la sécrétion de l'AVP/ADH [17]. L'hyponatrémie résulte d'une augmentation de l'eau corporelle causée par une combinaison d'apport hydrique excessive et d'une altération de l'excrétion de l'eau libre. Il peut être aggravé par une augmentation de l'excrétion urinaire de sodium déclenché par une expansion volémique et/ou une diminution de l'apport en sodium résultant de l'anorexie induite par l'hyponatrémie. Ainsi, pour retenir le diagnostic de SIAD, le patient ne doit pas avoir de signes d'hypervolémie (par exemple, œdème généralisé dû à une insuffisance cardiaque congestive, la cirrhose, ou syndrome néphrotique), d'hypovolémie (par exemple, l'hémorragie, la gastro-entérite, l'abus en diurétique, ou de déficit en aldostérone), d'insuffisance rénale ou d'insuffisance surrénale. La présence d'un état d'euvolémie clinique est considérée comme indispensable. L'osmolalité urinaire doit dépasser 100 mOsm par kilogramme d'eau sur une osmolalité plasmatique efficace basse. L'hypouricémie, le faible taux de l'urée sanguine et une natriurése supérieure à 40 mmol par litre chez les patients présentant une hyponatrémie suggèrent un SIAD sans pour autant être des critères diagnostiques formels [18]. En effet, un taux d'acide urique sérique inférieur à 238 μmol par litre [(4 mg/dl) en présence d'une hyponatrémie] à une valeur prédictive positive pour le diagnostic de SIAD de 73 à 100%. Une dilution de la natrémie de 10% s'accompagnant d'une dilution de 50% de l'uricémie par diminution de la réabsorption tubulaire d'acide urique. Ainsi, dans le cas difficile d'hyponatrémie survenue sous diurétique, une fraction d'extraction de l'acide urique supérieure à 12% permettrait d'identifier un SIAD [19]. Les critères formels pour le diagnostic du SIAD sont résumés dans le Tableau 2. Le type d'antidiurèse inappropriée de chaque patient est reproductible et reflète probablement un mécanisme pathogène distinct. Cependant, il ne semble pas en corrélation avec la maladie associée causale du SIAD et, en dehors de la production ectopique tumorale d'AVP/ADH, on sait peu de choses sur l'anomalie de base qui provoque les différents types d'antidiurése inappropriée. Physiologiquement, la relation entre ADH et osmolarité plasmatique est linéaire ce qui n'est pas systématiquement le cas dans le SIAD. Les travaux de Robertson [17, 20] ont permis de définir quatre types de SIAD en fonction du niveau, de la stabilité de la sécrétion d'HAD et de l'évolution de la sécrétion d'ADH par rapport à la natrémie après perfusion de sérum salé hypertonique (Tableau 3). Trois des quatre types d'antidiurése indiquent différentes anomalies dans la régulation osmotique de la sécrétion de l'AVP/ADH: erratique ou libération non régulée (type A), une “fuite” lente et constante (type B), et un abaissement de l'osmostat (type C). La mise en évidence du type D associant un tableau typique de SIADH et une ADH indétectable est à l'origine de la nouvelle appellation de SIAD [21] plutôt que de SIADH (sécrétion inappropriée d'ADH). L'osmorégulation de l'AVP/ADH semble être normale et l'antidiurése inappropriée résulte d'une anomalie au niveau du tubule collecteur rénal. Le tableau clinique et la réponse au traitement des patients atteints de différents types de SIAD peuvent également varier considérablement en fonction du type d'osmorégulateur défectueux. Cela est particulièrement vrai pour le type C (reset osmostat) répondant moins bien à la restriction hydrique, la perfusion de sérum salé hypertonique, ou aux antagonistes des récepteurs V2 car la sécrétion d'AVP/ADH augmente à mesure que l'hyponatrémie se corrige [17].

**Tableau 2:** Critères diagnostiques du syndrome d’antidiurèse inappropriée

Critère 1	Diminution de l'osmolalité efficace (<275 mOsm / kg d'eau)
Critère 2	Osmolalité urinaire> 100 mOsm / kg d'eau malgré l’hypotonicité
Critère 3	Euvolémie clinique (volume extracellulaire normal même en orthostatisme)
Critère 4	Sodium urinaire> 40 mmol / l en régime alimentaire sodé normal
Critère 5	Fonctions thyroïdienne et surrénalienne normales
Critère 6	Aucune utilisation récente de diurétiques
Critère 7	Acide urique plasmatique < 238 µmol/l
Critère 8	Urée plasmatique <3.5 mmol/l
Critère 9	Excrétion fractionnelle de sodium> 1% ; excrétion fractionnelle de l'urée> 55%
Critère 10	Pas de correction de l'hyponatrémie après perfusion de sérum salé isotonique
Critère 11	Correction de l'hyponatrémie par la restriction hydrique
Critère 12	Résultat anormal du test de charge en eau (<80% de l'excrétion de 20 ml d'eau par kg de poids corporel sur une période de 4 heures), ou dilution de l’urine insuffisante (<100 mOsm / kg d'eau)
Critère 13	AVP/ADH plasmatique élevée malgré la présence d'hypotonie et d’une euvolémie clinique

**Tableau 3:** Les différents types de syndrome d'antidiurèse inapproprié (SIAD)

	Type A	Type B	Type C	Type D
Fréquence (%)	37%	16%	33%	14%
Terminologie	Libération erratique d’AVP	Fuite permanente non suppressible d’AVP	Déplacement (abaissement) du seuil Reset osmostat syndrome	Antidiurèse hypovasopressinémique
Mécanisme	Sécrétion au hasard, indépendante du facteur osmotique. Variation rapide de stimuli non osmotique	Sécrétion ectopique et constante. Libération neuro hypophysaire continue	Osmorégulation préservée mais libération prématurée de l’AVP/ADH	Pas d’anomalie de sécrétion de l’AVP. Mutation activatrice V2R
Taux d’ADH	Fluctuant mais modéré à élevé	Modéré et constant	Bas	Bas à nul
Sécrétion d’ADH après perfusion de SSH	Non modifiée	Faible augmentation quand Na > 140 mmol/l	Augmentation linéaire mais pour Na < 140 mmol/l	Augmentation linéaire quand Na > 140 mmol/l
Etiologies	Cancer	Diverses	Diverses	Génétique

**Diagnostic différentiel:** il est extrêmement important de faire la part entre le SIAD et le *cerebral salt wasting syndrome* (CSWS), le facteur de confusion étant dans les deux cas l'existence d'une hyponatrémie associée à une natriurèse élevée (Tableau 4). Le CSWS, ou syndrome de perte de sel, est une hyponatrémie par fuite rénale de sodium (balance sodée négative) et une hypovolémie en rapport avec une sécrétion excessive de facteur atrial natriurétique (FAN). Le FAN est responsable de l'augmentation de la diurèse ainsi que de la natriurése, de la suppression de la sécrétion de rénine et d'aldostérone et produit une vasodilatation. Sa libération physiologique est liée à la distension auriculaire et est modulée par le système nerveux central. Au niveau cérébral, la concentration de ce peptide est 10 000 fois inférieure à celle du muscle cardiaque et insuffisante à elle seule pour rendre compte du CSWS. On considère donc qu'une lésion intracrânienne peut perturber la régulation centrale de la libération de FAN, dont l'excès est responsable du CSWS. On attribue à ce syndrome un rôle prépondérant dans l'apparition de l'hyponatrémie des patients victimes d'une hémorragie méningée anévrysmale, d'autant que l'hypovolémie y est fréquente et qu'une balance sodée négative précède l'apparition de l'hyponatrémie [22-24]. Il existe une corrélation entre le score de gravité de l'hémorragie sous-arachnoïdienne et l'élévation des concentrations sériques de FAN [25]. Le CSWS a également été observé au cours de traumatismes craniocérébraux, de méningites carcinomateuses, en cas de tumeurs cérébrales primitives ou secondaires ainsi qu'au décours de la chirurgie hypophysaire [22]. Cette entité, étiquetée cerebral salt wasting syndrome car décrite initialement après chirurgie cérébrale a été rebaptisée SWS [26] après la publication de cas secondaires à des pathologies extrachirurgicales (méningite, encéphalite) et extracérébrales (médicaments, pneumonies). Le diagnostic différentiel entre SIAD et CSWS est capital, compte tenu d'implications thérapeutiques radicalement opposées [27, 28]. L'élément clé est l'appréciation de la volémie [22, 23, 27, 29]. Les techniques isotopiques de mesure de la volémie réalisables au lit du patient représentent donc une aide de premier choix [30]. La prise en charge thérapeutique sera fondée sur la restriction hydrique pour le SIAD et sur des apports hydrosodés très larges pour le CSWS.

**Tableau 4:** Critères différentiels entre syndrome d'antidiurèse inapproprié (SIAD) et cerebral salt wasting syndrome (CSWS)

	SIAD	CSWS
**Déshydratation**	Absente	Présente
**Poids**	Normal ou augmenté	Diminué
**Volémie**	Augmentée	Diminuée
**Balance sodée**	Normal ou variable	Diminuée
**Pression veineuse centrale**	Normale ou augmentée	Diminuée
**Hématocrite**	Basse ou normale	Augmentée
**Osmolalité**	Diminuée	Augmentée ou normale
**Protidémie**	Normale	Augmentée
**Natriurése**	Augmentée	Très augmentée
**Uricémie**	Diminuée	Normale ou augmentée

**Le syndrome d'antidiurèse inappropriée néphrogénique (NSIAD):** le tableau est évocateur d'un SIADH mais avec un dosage plasmatique d'AVP/ADH bas ou effondré. Sa fréquence n'est pas connue, mais pourrait ne pas être rare. Les précédentes études de patients avec SIAD ont rapporté des taux variables de sécrétion d'AVP/ADH, en particulier en réponse à une charge et/ou une restriction hydrique [31]. Environ 10 à 20% des patients atteints avaient des taux d'ADH inférieurs à la limite de détection par dosage radio-immunologique. Ainsi, certains de ces patients pourraient en fait être atteints du NSIAD [31]. Bien que de nombreuses mutations inactivatrices du V2R liées à l'X ont été décrits au cours du diabète insipide néphrogénique [32], quelques rares patients définis comme un SIADH chronique avec des niveaux d'AVP indétectables et une mutation activatrice gain-de-fonction de l'V2R ont été récemment signalés [12]. La mutation R137H est à l'origine du diabète insipide néphrogénique, et les mutations R137L et R137C causent le NSIAD. A la lumière de ces résultats, il a été suggéré de référencer toutes les conditions SIADH-like comme des NSIAD. En effet, le séquençage de l'ADN du gène V2R des patients atteints a permis d'identifier des mutations faux-sens dans le codon 137 avec réarrangement de l'arginine à la cystéine ou à la leucine. Le mécanisme par lequel ces mutations faux-sens activent constitutivement la V2R nécessite d'autres études plus approfondies. Ainsi, le séquençage du gène V2R est recommandé pour identifier les mutations spécifiques. Ces mutations liées à l'X, activatrices des récepteurs V2 de l'AVP/ADH, ont été retrouvées chez des enfants, définissant le SIAD néphrogénique (NSIAD) [33] mais probablement aussi chez l'adulte et dans les deux sexes comme rapporté par Decaux [34]. D'autres patients pourraient à l'avenir être identifiés avec un phénotype NSIAD sans mutations de V2R, mais plutôt une mutation activatrice de l'AQP-2 ce qui suggèrerait la présence de défauts supplémentaires dans cette cascade de signalisation. Le traitement du NSIAD n'est pas défini. La restriction hydrique améliore la natrémie et l'osmolalité, mais limite l'apport calorique notamment chez les nourrissons. Les agents qui agissent en aval de la V2R, comme la demeclocycline ou le lithium, pourraient antagoniser les récepteurs constitutivement activés, mais présentent des effets secondaires potentiellement limitants. Un traitement par l'urée pour induire une diurèse osmotique peut être proposé [12, 16, 35] comme décrite pour la première fois par Decaux *et al.* au cours du traitement du SIADH chronique chez les adultes [36, 37]. A la posologie de 15g deux fois par jour, elle accroît la diurèse (par augmentation de la charge osmotique) et inhibe la natriurèse tant que la natrémie reste inférieure à 130 mmol/l [38]. L'utilisation de l'urée ne nécessite pas la surveillance des apports sodés et n'induit pas de déplétion potassique. Ses effets secondaires sont rares (principal inconvénient est lié à son goût qui peut être amélioré en le mélangeant avec du jus de fruits), même après un traitement de plusieurs années. Elle a ainsi pu être utilisée chez des patients de neurochirurgie, permettant une élévation de la natrémie d'environ 8 mmol/l après un à deux jours de traitement [39]. Associés à une restriction hydrique, l'administration d'urée comporte un risque d'hypernatrémie si le patient ne peut boire (coma) ou si coexiste un trouble de la soif. Les inhibiteurs des récepteurs à la vasopressine (vaptans) interfèrent directement avec le mécanisme du SIAD en empêchant l'ADH de stimuler les récepteurs V2 du tubule collecteur. Cinq molécules sont connues (lixtvaptan, satavaptan, mozavaptan, conivaptan, tolvaptan). Le tolvaptan per os (seule molécule disponible en France) a démontré son efficacité sur l'hyponatrémie dans une population hétérogène (cirrhose, insuffisance cardiaque, SIAD) (études SALT-1 et -2) [40] et sa bonne tolérance au long cours (deux ans ; étude SALTWATER) [41]. Les vaptans augmentent en moyenne la natrèmie de +5,27 mmol/l (4,27-6,26) précocement et de 3,49 mmol/l (2,56-4,41) tardivement par rapport au placebo [42]. Cependant, à l'heure actuelle, il existe plus d'arguments (notamment économiques, mais aussi de tolérance et d'efficacité) en faveur de l'urée comme traitement de première intention dans l'hyponatrémie secondaire au SIAD, l'utilisation des vaptans devant être réservée en cas d'échec ou d'intolérance à l'urée. Enfin, le seul traitement définitif du NSIAD est l'élimination de la cause sous-jacente et notamment ceux qui sont dus aux médicaments.

## Conclusion

Le NSIAD est une situation clinique SIADH-like à ADH plasmatique basse due à une mutation gain-de-fonction liée à l'X se traduisant par des changements faux-sens d'acides aminés dans le V2R aboutissant à une activation constitutive du récepteur.

### Etat des connaissances actuelles sur le sujet

- Peu de choses connues;
- Physiopathologie complexe;
- Pathologie assez fréquente en pratique médicale.

### Contribution de notre étude à la connaissance

- Revue récente et globale de la littérature;
- Physiopathologie expliquée et claire;
- Exemples de pathologies diverses et résumé des critères diagnostiques.

## Conflits d’intérêts

Les auteurs ne déclarent aucun conflit d'intérêts.
